# A rapid, simple and sensitive LC-MS/MS method for lenvatinib quantification in human plasma for therapeutic drug monitoring

**DOI:** 10.1371/journal.pone.0259137

**Published:** 2021-10-26

**Authors:** Martina Zanchetta, Valentina Iacuzzi, Bianca Posocco, Giorgia Bortolin, Ariana Soledad Poetto, Marco Orleni, Giovanni Canil, Michela Guardascione, Luisa Foltran, Valentina Fanotto, Fabio Puglisi, Sara Gagno, Giuseppe Toffoli

**Affiliations:** 1 Experimental and Clinical Pharmacology Unit, Centro di Riferimento Oncologico di Aviano (CRO) IRCCS, Aviano, Italy; 2 Department of Chemical and Pharmaceutical Sciences, University of Trieste, Trieste, Italy; 3 Doctoral School in Pharmacological Sciences, University of Padova, Padova, Italy; 4 Unit of Medical Oncology and Cancer Prevention, Centro di Riferimento Oncologico di Aviano (CRO) IRCCS, Aviano, Italy; 5 Department of Medicine (DAME), University of Udine, Udine, Italy; Cairo University, EGYPT

## Abstract

Lenvatinib (LENVA) is an oral antineoplastic drug used for the treatment of hepatocellular carcinoma and thyroid carcinoma. LENVA therapeutic drug monitoring (TDM) should be mandatory for a precision medicine to optimize the drug dosage. To this end, the development of a sensitive and robust quantification method to be applied in the clinical setting is essential. The aim of this work was to develop and validate a sensitive, rapid, and cost-effective LC-MS/MS method for the quantification of LENVA in human plasma. On this premise, sample preparation was based on a protein precipitation and the chromatographic separation was achieved on a Synergi Fusion RP C18 column in 4 min. The method was completely and successfully validated according to European Medicines Agency (EMA) and Food and Drug Administration (FDA) guidelines, with good linearity in the range of 0.50–2000 ng/mL (R≥0.9968). Coefficient of variation (CV) for intra- and inter-day precision was ≤11.3% and accuracy ranged from 96.3 to 109.0%, internal standard normalized matrix effect CV% was ≤2.8% and recovery was ≥95.6%. Successful results were obtained for sensitivity (signal to noise (S/N) ratio >21) and selectivity, dilution integrity (CV% ≤ 4.0% and accuracy 99.9–102%), and analyte stability under various handling and storage conditions both in matrix and solvents. This method was applied to quantify LENVA in patient’s plasma samples and covered the concentration range achievable in patients. In conclusion, a sensitive and robust quantification method was developed and validated to be applied in the clinical setting.

## Introduction

Lenvatinib (LENVA) is an oral multikinase inhibitor with antiangiogenic and antiproliferative properties, that targets VEGF receptors 1–3, FGF receptors 1–4, PDGF receptor α, RET, and KIT [1]. It was approved by Food and Drug Administration (FDA) and European Medicines Agency (EMA) as monotherapy for the treatment of differentiated thyroid carcinoma (DTC), and hepatocellular carcinoma (HCC) [2, 3]. FDA approved LENVA also for the treatment of advanced renal cell carcinoma [2].

Oral drugs in chronic administration, such as LENVA, are becoming a new strategy in cancer therapy and Therapeutic Drug Monitoring (TDM) should be mandatory to modulate the drug dosage since intra- and inter-patients variations in drug plasma concentrations can occur. Many reports indicate that, for chronically administered oral drugs, minimum drug concentration at the steady-state (C_min_) is one of the best pharmacokinetic (PK) parameters to be used for adjusting the drug dosage in cancer patients [4].

In addition to being a good candidate for TDM, LENVA has no active metabolites, which makes PK analysis easier as metabolites determinations are devoid of clinical utility. Concerning the exposure-response relationship of this drug, Hata et al. observed that HCC patients with a C_min_ higher than 42.68 ng/mL had a better objective response rate (ORR) than those with lower C_min_ [5]. Nagahama and colleagues reported a C_min_ threshold for LENVA toxicity of 88 ng/mL in Japanese DTC patients [6].

An exploratory target plasma concentration (C_min_ of 51.5 ng/mL) has been suggested for LENVA TDM [4], but due to the small number of studies still reported, further validations are required.

To apply TDM in the clinical routine, the development of sensitive and robust quantification method is essential. At the best of our knowledge, the published liquid chromatography-tandem mass spectrometry (LC-MS/MS) methods for LENVA quantification in human plasma are reported in Table 1.

**Table 1:** Comparison between the proposed and previously reported LC-MS/MS methods for quantification of LENVA in human plasma.

Ref.	Analyte	Sample Volume (μL)	Extraction Method	Runtime (min)	Linearity Range
[7]	LENVA and 4 metabolites (M1: Decyclopropylation; M2:demethylation; M3: N-oxidation; M5 O-dearylation), ER-227326 (IS)	250	PP with supernatant evaporation and re-dissolution	21	0.25–50 ng/mL
[8]	LENVA and LENVA-D_4_ (IS)	200	LLE	8	10.20–501.6 pg/mL
[9]	LENVA, propanolol (IS)	250	PP with supernatant evaporation and re-dissolution	15	9.6–200 ng/mL
[10]	alectinib, cobimetinib, LENVA, nintedanib, osimertinib, palbociclib, ribociclib, vismodegib, vorinostat, alectinib-D_8_(IS), LENVA-D_5_ (IS), nintedani-^13^C,D_3_(IS), osimertinib-^13^C,D_3_ (IS), palbociclib-D_8_ (IS), ribociclib-D_6_ (IS), vismodegib-^13^C_7_ (IS), vorinostat-^13^C_6_ (IS), cobimetinib-^13^C_6_ (IS)	50	PP	4	10–200 ng/mL
[11]	LENVA and LENVA-D_4_ (IS)	100	SPE	6	0.2–1000 ng/mL
[12]	Axitinib, LENVA, afatinib, bosutinib, cabozantinib, dabrafenib, osimertinib, ruxolitinib, nilotinib, trametinib, afatinib-D_6_ (IS),bosutinib-D_9_(IS), dabrafenib-D_9_(IS), LENVA-D_5_ (IS), osimertinib-^13^C,D_3_ (IS), trametinib-^13^C,D_6_ (IS), axitnib-^13^C,D_3_ (IS), cabozantinib-D_4_ (IS), nilotinib-D_6_ (IS) and ruxolitinib-D_4_ (IS)	50	PP	7	2–500 ng/mL
[13]	sorafenib, LENVA, apatinib, sorafenib D_3_ (IS), LENVA D_4_ (IS), apatinib D_8_ (IS)	100	PP with dilution in MP A	3.5	1.25–40 ng/mL
The proposed method	LENVA and LENVA-D_4_ (IS)	100	PP	4	0.5–2000 ng/mL

IS: internal standard; LLE: liquid-liquid extraction; MP A: mobile phase A; PP: protein precipitation; SPE: solid-phase extraction.

Dubbelman et al. [7] quantified LENVA and four metabolites in three different matrices (human plasma, urine, and feces) and LENVA alone in whole blood. The validated analytical range in plasma was 0.25–50 ng/mL, which was lower than the target C_min_ (51.5 ng/mL). Moreover, this method is time-consuming (twenty-one min of runtime and a sample preparation based on protein precipitation (PP) followed by supernatant evaporation and re-dissolution steps) and requires a large sample volume (250 μL).

Ogawa-Morita and colleagues partially modified Dubbelman’s method extending the linear range (9.6–200 ng/mL) [9] and reducing analysis run time (15 min) but sample preparation remained time-consuming.

Srikanth et al.‘s method [8] was relatively fast (8 min), but required a large sample volume (200 μL), a complex sample preparation (liquid-liquid extraction-LLE) and the validated analytical range (10.20–501.6 pg/mL) was below the target LENVA C_min_.

Recently, Sueshige and colleagues [11] developed a LC-MS/MS quantification method for LENVA with a reduced sample volume (100 μL), short runtime (4 min), and adequate range for TDM application (0.2–1000 ng/mL). Nonetheless, the sample preparation was based on solid-phase extraction (SPE) that could make this method complex and laborious.

In the literature three other methods were reported for LENVA quantification with other 8, 9 or 2 kinase inhibitors, respectively [10, 12, 13]. These methods overcome the above-mentioned limitations: they required a small sample volume (50/100 μL), had a fast chromatographic run (between 3.5 and 7min) and a simple sample preparation (PP). Concentration ranges were suitable for the target C_min_ of 51.5 ng/mL with the exception of the method developed by Ye et al. [13] (1.25–40 ng/mL).

However, the quantification of other kinase inhibitors was not necessary for our purpose and we considered of interest the possibility to have a wider concentration range in order to obtain a method useful not only for LENVA C_min_ monitoring in patients with HCC but also for PK investigations in patients affected by other pathologies and treated with LENVA at higher doses (*e*.*g*., LENVA is administered at the dose of 24 mg/day in patients with DTC). For these reasons, we developed and validated according to EMA and FDA guidelines, a LC-MS/MS method for the quantification of LENVA in a wide concentration range to be used for cancer patients’ plasma samples. It required a relatively low sample volume, an easy and quick sample processing based on PP, and a reasonable runtime.

## Material and methods

### Chemicals and reagents

The analytical standard of LENVA (batch: 6-JTN-66-1; purity: 98%) and LENVA-D_4_ (batch: CS-SI-AAA-0949-01, chemical and isotopic purity: 99.18% and 99.48%, respectively) were supplied by Toronto Research and Chemical Inc. (North York, Ontario, Canada) and Clearsynth Labs Ltd. (Mumbai, India), respectively. Formic acid and LC-MS grade isopropanol were provided by Merck-Sigma (Milano, Italy), while LC-MS grade methanol was supplied by Carlo Erba (Milano, Italy).

“Type 1” ultrapure water was produced at our laboratory by a Milli-Q^®^ IQ 7000 system (Merck). Plasma/K-EDTA from healthy donors to prepare standard calibration curves and quality control samples (QCs) was provided by the Transfusion Unit of our Institute.

### Standard solutions preparation

Stock solutions of LENVA and LENVA-D_4_ were prepared in DMSO at the concentration of 1 mg/mL. Two different stock solutions were obtained for LENVA: one for the preparation of the calibration curve and the other for QCs. To obtain the working solutions for the calibration curve (from A to H), the stock solution of LENVA was diluted with methanol to achieve the final concentrations of: 40.0, 20.0, 10.0, 2.00, 0.80, 0.30, 0.06 and 0.01 μg/mL. The same procedure was applied also to obtain the working solutions for the QCs with a final concentration of 30.0, 1.50 and 0.03 μg/mL. IS stock solution was also diluted in acidified methanol with 0.10% formic acid to obtain the final concentration of 50.0 ng/mL. This solution was directly used to perform protein precipitation during sample processing.

### Calibration curve and QCs sample preparation

Every day, an eight-point calibration curve (A to H) and triplicates of each QC concentration were freshly prepared in plasma. A blank sample (plasma processed without IS) and a zero-blank sample (plasma processed including IS) were analyzed before each analysis. The preparation of calibrators and QCs samples was conducted as follows: 95 μL of pooled blank human plasma were added with 5 μL of working solutions (dilution 1:20) and vortex-mixed for 10 s, obtaining the final concentrations of 2000, 1000, 500, 100, 40.0, 15.0, 3.00 and 0.50 ng/mL for the calibration curve and 1500, 75.0 and 1.50 ng/mL for QCs. One hundred μL-aliquots of QCs were prepared and stored at -80 °C to allow the assessment of analytes long-term stability and to be used as controls in future analyses.

### Samples handling

A hundred μL of each calibrator and QC (95 μL plasma + 5 μL working solution) or patient’s plasma sample were added with 500 μL of cold IS working solution to precipitate plasma proteins, vortex-mixed for 10 s and centrifuged for 25 min at 16200 g at 4 °C. Finally, the clean supernatant was transferred into auto-sampler glass vials and 4 μL were injected in the LC-MS/MS apparatus for the analysis.

### Chromatography and mass spectrometry conditions

The method was developed and validated using a SIL-20AC XR autosampler and Nexera LC-20AD UFLC XR pumps (Shimadzu, Tokyo, Japan) coupled with an API 4000 triple quadrupole spectrometer (AB SCIEX, Massachusetts, USA). The extracted samples were injected into a Synergi-Fusion C18 column (4 μm, 80 Å, 30 x 2 mm I.D.) coupled with a Security Guard Cartridge (Fusion, C18, 4 x 2.0 mm), both produced by Phenomenex (Castel Maggiore (BO), Italy). The oven temperature was set at 50 °C. The mobile phase was composed by ultrapure water plus 0.10% formic acid (v/v) (eluent A) and methanol/isopropanol (90:10, v/v) with 0.10% of formic acid (v/v) (eluent B).

The spectrometer was equipped with an electrospray source (TurboIonSpray^®^ probe) and worked in positive ion mode. Compound and source MS parameters were optimized by directly injecting a 100 ng/mL solution of LENVA and IS with a flow rate of 20.0 μL/min. Analyst software (1.6.3 version) was used for both data acquisition and quantification.

### Validation procedures

A full validation of the proposed method was conducted according to FDA and EMA guidelines on bioanalytical method validation [14, 15] performing the below described evaluations.

#### Recovery, matrix effect and selectivity

Recovery was determined by preparing in quintuplicate the QCs at each concentration level and comparing the analyte peak area ratio of plasma samples spiked both before and after protein precipitation.

Matrix effect was evaluated with different strategies during method development and validation. First, a qualitative estimation was performed through post-column infusion using a standard solution of LENVA in methanol with 0.10% formic acid (v/v) at a concentration of 100 ng/mL with a flow rate of 20 μL/min. Then, a quantitative evaluation of matrix effect was performed using 6 replicates of the QCs (QCL, QCM, QCH) and IS using matrix from 6 different donors (3 females and 3 males) and by comparing the peak area ratio of post extraction QCs (QC working solution added to extracted plasma sample) or IS with those obtained from QCs or IS prepared in pure methanol. Moreover, the IS normalized matrix effect was calculated as the ratio between the matrix effect of analyte and the matrix effect of IS. The coefficient of variation (CV%) of IS normalized matrix effect should not be greater than 15%.

Selectivity was investigated by analyzing 6 blank human plasma samples obtained from 6 different donors (3 females and 3 males). These samples should be free of interference at the retention time of the analyte of interest (a response lower than 20% of the lower limit of quantification (LLOQ) for LENVA and lower than 5% for the IS).

#### Linearity and sensitivity

Linearity was assessed by preparing 8 calibration curves, which were freshly processed during 8 different working days. Calibration curves were obtained using a weighted quadratic regression model (1/x^2^). The Pearson’s determination coefficient R was calculated for each calibration curve and the comparison between the nominal and back-calculated concentrations of each calibrator (expressed as accuracy) was checked. At least 75% of the calibrators, including the LLOQ and the upper limit of quantification (ULOQ), had to be within 85–115% of the nominal concentration (80–120% at the LLOQ).

Sensitivity is defined by the LLOQ, which is the lowest concentration that could be measured with a precision within 20%, accuracy between 80% and 120%, and a signal-to-noise ratio (S/N) ≥ 5. The LLOQ of the present method was verified by analyzing precision, accuracy, and S/N ratio obtained from 6 samples of pooled blank human plasma added with “H” (i.e. the least concentrated) working solution.

#### Intra- and inter-day precision and accuracy

Intra-day precision and accuracy were determined during a single working day by analyzing 6 replicates of the LLOQ and each QC concentration, while inter-day precision and accuracy were assessed on 5 different working days, analyzing 3 replicates of the LLOQ and each QC concentration with a calibration curve freshly prepared every day. The measured concentrations had to be within ±15% of the nominal value with a CV% ≤ 15% for at least 67% of the QCs at each concentration level in each run (only one QC for each concentration level could be excluded). For the LLOQ samples, the measured concentration had to be within ±20% and have a CV% ≤ 20%.

#### Stability and dilution integrity

Bench-top and long-term stability was assessed for LENVA using QCs prepared in triplicate at each concentration (QCL, QCM, QCH): bench-top stability in plasma was investigated after 4 h at room temperature; the post-processing stability of the extracted QCs was evaluated in autosampler set at 4 °C re-analyzing the samples 20, 44, 70 and 94 h after the first injection; freeze/thaw stability was assessed by analyzing 3 freshly prepared aliquots of each QCs concentration, and then again after one, two and three freeze/thaw cycles. Long-term stability was investigated both in plasma, to assess patient samples stability after storage at -80 °C, and in solvent (methanol or DMSO) to assess working solutions or stock solutions stability after storage at -20 °C and -80 °C, respectively. Stability tests were considered verified if the samples tested did not exceed ±15% from the nominal concentrations at each QCs concentration.

Dilution integrity was evaluated on a plasma sample at a LENVA concentration of 3000 ng/mL, using 1:10 and 1:100 dilution factors, using pooled plasma as a diluting agent. Each dilution factor was tested in quintuplicate and the measured concentrations had to be within ±15% of the nominal value with a CV% ≤ 15%.

#### Incurred sample reanalysis

Reproducibility or incurred sample reanalysis (ISR) was verified by repeating the analysis of a subset of patients’ samples (n = 14) in separate runs. The two analyses could be considered equivalent if the percentage difference [expressed as: (repeat-original)*100/mean] between the first and the second concentration measured was within ±20% for at least 67% of the samples.

### Application of the method to patient samples

The proposed method was developed in the context of an analytical cross-validation study (CRO-2018-83) ongoing at the National Cancer Institute (*Centro di Riferimento Oncologico*—CRO) of Aviano. The study aims at assessing the reliability of innovative analytical methods based on Dried Blood Spot (DBS) for the quantification of several anticancer drugs, including LENVA, by comparing such methods with the Gold Standard LC-MS/MS methods in plasma. The proposed method was used as a reference method to quantify LENVA concentration in plasma samples from HCC patients recruited in the study from October 2020 to July 2021. Patients entered the study according to the following eligibility criteria: 1) to be treated with LENVA according to the routine clinical practice at any dose and any treatment cycle; 2) age ≥ 18 years; 3) life expectancy > 3 months; 4) provide a signed written informed consent. Blood samples were collected into 2.7 mL K-EDTA tubes after at least 7 days of treatment (time necessary to reach the steady-state) at each clinical visit of patients (about every month). Plasma was obtained immediately by centrifugation of the blood samples at 2600 g for 10 min at 4 °C. The obtained plasma was split into three independent aliquots and stored at -80 °C until analysis.

### Ethics statement regarding patients’ samples

The analytical cross-validation study (protocol code: CRO-2018-83) was approved by the local ethics committee (*Comitato Etico Unico Regionale*- C.E.U.R.) and is conducted according to the Declaration of Helsinki principles [16]. Patients were informed by the oncologist about the analytical study during their visits and were recruited only after the signature of written informed consent.

## Results and discussion

### LC-MS/MS conditions

**Fig 1:**
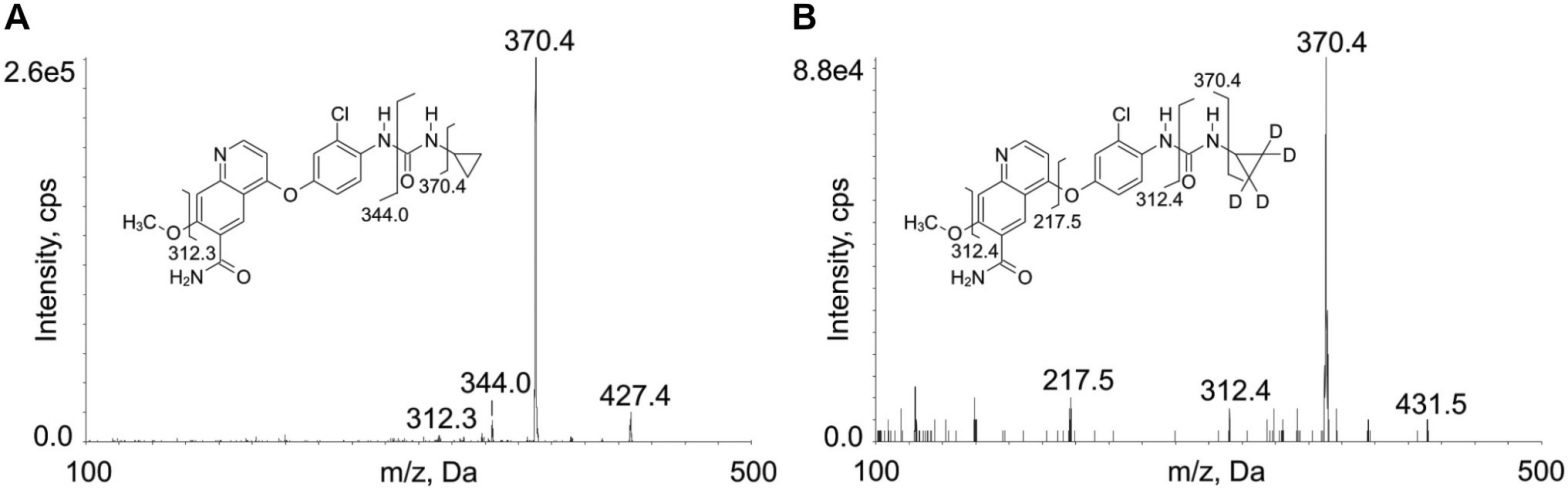
MS/MS mass spectra with chemical structures and identification of the fragment ions of LENVA (A) and internal standard (LENVA-D_4_) (B); spectra were recorded with CE = 40 V. LENVA fragment at 312.3 *m/z* derived from 344.0 *m/z* fragment by the loss of methoxy group.

The electrospray ionization source (ESI) was set in positive ion mode, thus LENVA and IS mainly produced protonated molecules [M+H^+^] for the presence of amino groups. Source dependent parameters were optimized as follows: the temperature was set at 550°C, nebulizer gas pressure was 50 psi and that of the heater gas was of 40 psi (zero air), curtain gas flow was regulated at a pressure of 35 psi and that of the collision gas (CAD) at 6 (nitrogen), ion spray voltage was 5500 V. The most intense daughter ion for LENVA, which *m/z* was 370.4, was used as a quantifier transition, while the fragment ions used as qualifiers were 312.2 *m/z* and 344.0 *m/z*. The quantification of the IS signal was conducted using the following transition: 370.4 *m/z* as a quantifier, while 312.4 *m/z* and 217.5 *m/z* as qualifiers. The fragmentation patterns obtained within the collision cell are represented in Fig 1 and reported in Table 2 along with the optimized compound-dependent parameters.

**Table 2:** Optimized compound-dependent parameters of LENVA and LENVA-D_4_ (IS).

Compound	Q1^a^ (m/z)	DP^b^ (V)	EP^c^ (V)	Q3^d^ (m/z)	CE^e^ (V)	CXP^f^ (V)
**LENVA**	427.4	140	10	370.4	37	10
				312.2	60	10
				344.0	40	10
**LENVA-D4**	431.5	120	10	370.4	40	10
				312.4	60	10
				217.5	30	10

^a^first quadrupole mass;

^b^declustering potential;

^c^entrance potential;

^d^third quadrupole mass;

^e^collision energy;

^f^collision cell exit potential.

**Fig 2:**
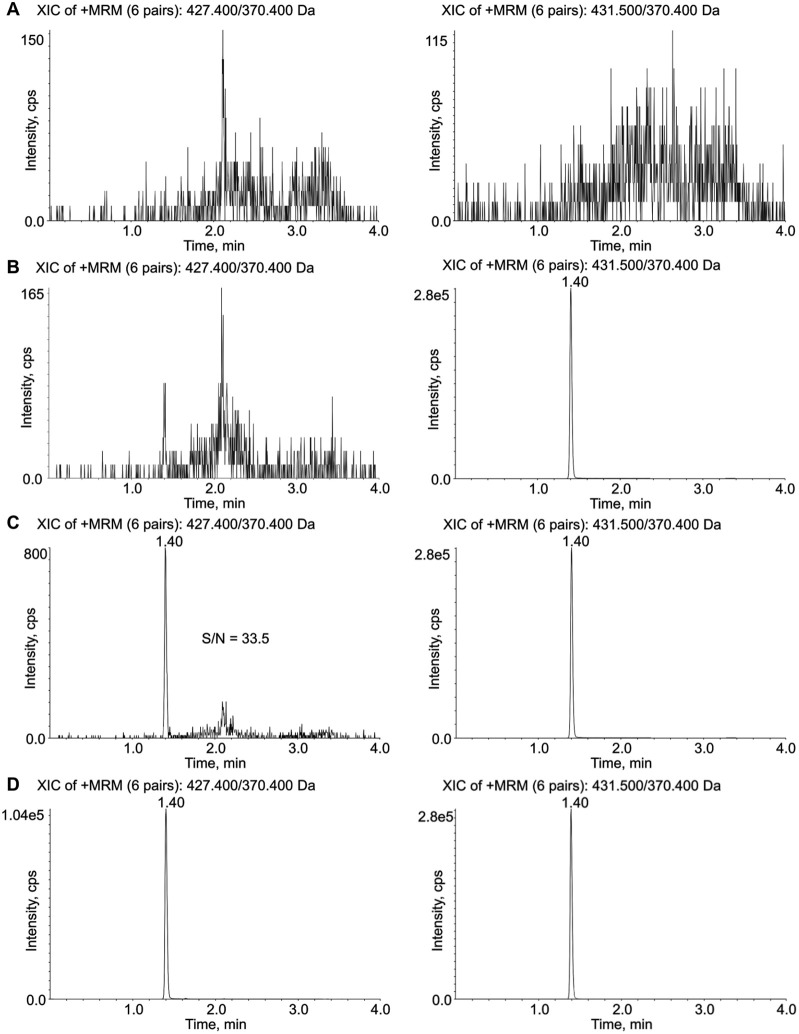
MRM chromatograms for LENVA (left panels) and internal standard (right panels). A: blank plasma sample; B: blank plasma sample with IS (50 ng/mL); C: LLOQ (0.50 ng/mL) with S/N value; D: MRM chromatograms of plasma sample from a patient treated with 12 mg/day LENVA and showing a drug concentration of 99.6 ng/mL.

Different mobile phases were tested (e. g., acetonitrile with 0.10% formic acid (v/v) or methanol with 0.10% formic acid (v/v) both tested alone or mixed with isopropanol) and the best results in terms of peak shape and sensitivity were obtained with methanol/isopropanol (90:10, v/v) with 0.10% of formic acid (v/v) as eluent B and ultrapure water plus 0.10% formic acid (v/v) as eluent A. The chromatographic analysis was obtained using a flow rate of 0.60 mL/min, setting column temperature at 50 °C and applying the following gradient: the percentage of eluent B (methanol/isopropanol (90:10, v/v) with 0.10% of formic acid (v/v), eluent A: ultrapure water plus 0.10% formic acid (v/v)) was increased from the starting condition of 5% to 98% in 1.50 min, and then kept constant for 1.15 min to ensure an efficient-column washing; the initial condition was then restored in 0.10 min, and the column was re-equilibrated for 1.25 min. The total run time was 4.00 min. Fig 2 displays typical SRM chromatograms of plasma samples: an extracted blank plasma sample (Fig 2A), a zero blank sample containing IS only (Fig 2B), an extracted plasma sample at the LLOQ (Fig 2C), and a sample from a patient collected 4.5 hours after drug intake (dose 12 mg) with a measured LENVA concentration of 99.6 ng/mL (Fig 2D). As shown from the figure, the analyte was rapidly eluted, with a retention time of 1.40 min.

### Validation procedures

Recovery, matrix effect and selectivity. The percentage of LENVA recovery resulted high, ≥ 95.6% (range from 95.6 to 102, CV% ≤ 4.6), and reproducible over the concentrations ranges tested (Table 3). These percentages are the highest (89.5%) among the published methods (Table 1) based on the same sample treatment (PP) [7, 9, 12, 13].

**Table 3:** Recovery and matrix effect (ME) results of LENVA and LENVA-D_4_ in human plasma samples.

Compound	Nominal concentration (ng/ml)	Mean Recovery (%)±SD	CV%	Mean ME (%)±SD	CV%
LENVA	1.50	95.6±4.3	4.5	157 ±3.9	2.5
	75.0	97.8±4.5	4.6	-	-
	1500	102 ±1.6	1.6	136 ±3.1	2.3
LENVA-D_4_	50.0	-	-	125 ±7.0	5.6

Both the qualitative test of post-column infusion and the quantitative analysis obtained from the ratio between the analytes peak area in the presence of matrix (single donor plasma) and the peak area in the absence of matrix (methanol) using QCs, demonstrated the presence of a matrix effect. In fact, an enhancement of extracted ions signals (XIC) was detected at the retention time of the analytes, compared to those obtained in the pure solvent, as reported in Table 3. A variability on the estimated matrix effect (%ME) for LENVA was observed according to the concentration level (136 for QCH and 157 for QCL), whereas, within the concentration levels, results were highly reproducible with a %CV always < 2.5%. The %ME for LENVA-D_4_ resulted 125% with a CV% < 5.6%. The IS normalized matrix effect was 1.27±0.03 (SD) with a CV% ≤ 2.8% for QCL and 1.09±0.02 (SD) for QCH with a CV% ≤ 2.1. These data are slightly higher than previously published methods based on PP but with a much lower CV% [10, 12, 13]. The obtained CV% values were lower than the guidelines acceptance criteria (<15%) and so the matrix effect was considered negligible in affecting analyses results.

The selectivity of the proposed method was proved by analyzing 6 blank plasma samples from 6 different donors: no interference was detected at the retention time of the analyte (Fig 2A).

#### Linearity and sensitivity

The linearity of the method was demonstrated over the selected concentrations (2000, 1000, 500, 100, 40.0, 15.0, 3.00 and 0.50 ng/mL) preparing 8 calibration curves: the mean R value obtained was 0.999±0.001. Moreover, the calculated accuracy was between 95.9 and 105%, and precision was within 5.0%. In Table 4 the complete list of accuracy and precision data is reported.

**Table 4:** Precision (CV%) and accuracy %data of LENVA calibration curves in human plasma.

LENVA (N = 8)			
Nominal concentration(ng/mL)	Mean ± SD (ng/mL)	CV%	Accuracy%
0.50	0.50 ± 0.00	0.9	99.5
3.00	3.08 ± 0.15	5.0	103
15.0	15.7 ± 0.76	4.8	105
40.0	41.5 ± 0.82	2.0	104
100	102 ± 2.44	2.4	102
500	489 ±1 5.4	3.1	97.9
1000	959 ± 35.6	3.7	95.9
2000	1918 ± 89.2	4.7	95.9

Concerning the method sensitivity, the accuracy and precision (CV%) obtained for the 6 LLOQ samples (0.50 ng/mL) prepared in pooled blank human plasma were 98.4% and 10.0%, respectively. The S/N ratio obtained was always > 21 (Fig 2C).

#### Intra- and inter-day precision and accuracy

The intra-day precision and accuracy for LENVA, in 6 samples at each QC level and at the LLOQ, resulted to be ≤ 10.0% and between 96.3 and 109%, respectively. At the same time, inter-day precision and accuracy, tested on 5 different working days in triplicate for each QC level and the LLOQ, were ≤ 11.3% and between 98.0 and 108%, respectively.

The obtained data of intra- and inter-day precision and accuracy, reported in Table 5, complied with FDA and EMA requirements.

**Table 5:** Intra- and inter-day precision (CV%) and accuracy % obtained for LENVA.

**Intra-day (N = 6)**			
**Nominal concentration (ng/mL)**	**Mean ± SD (ng/mL)**	**CV%**	**Accuracy%**
0.50 (LLOQ)	0.49 ± 0.05	10.0	98.4
1.50	1.59 ± 0.03	1.7	106
75.0	81.7 ± 2.19	2.7	109
1500	1445 ± 34.5	2.4	96.3
**Inter-day (N = 15)**			
**Nominal concentration (ng/mL)**	**Mean ± SD (ng/mL)**	**CV%**	**Accuracy%**
0.50 (LLOQ)	0.50 ± 0.06	11.3	101
1.50	1.60 ± 0.08	4.9	107
75.0	80.6 ± 3.7	4.5	108
1500	1469 ± 97.9	6.7	98.0

#### Stability and dilution integrity

LENVA stability in plasma matrix was proved under handling and storage conditions. Bench-top stability was assessed after 4 h at room temperature with precision and accuracy within 10.9% and between 95.1% and 109%, respectively. LENVA was stable in plasma after 3 freeze (-80°C)/thaw cycles (precision and accuracy values were ≤ 6.3% and between 93.1 and 103%, respectively) and after 315 days of storage a -80 °C (precision ≤ 4.1% and an accuracy between 87.7 and 103%). These data are in line with those reported by the other methods presented in Table 1. With the proposed method, the long term stability in human plasma was tested and verified for a longer period (315 days *vs* 6 months). LENVA resulted stable also in extracted (deproteinized) plasma samples in autosampler set at 4 °C for 94 h, as proved by precision ≤ 4.6% and accuracy between 89.3 and 103%. The stability of LENVA in solvents was also assessed as follow: for at least 174 days both in DMSO stored at -80 °C (stock solution) and in methanol stored a -20 °C (working solutions) with a precision ≤ 5.6% and accuracy between 89.1 and 109% (S1 Table).

The dilution integrity of plasma samples was assessed at two dilution factors: 1:10 and 1:100 with a very good precision and accuracy (S2 Table).

#### Incurred sample reanalysis

**Fig 3:**
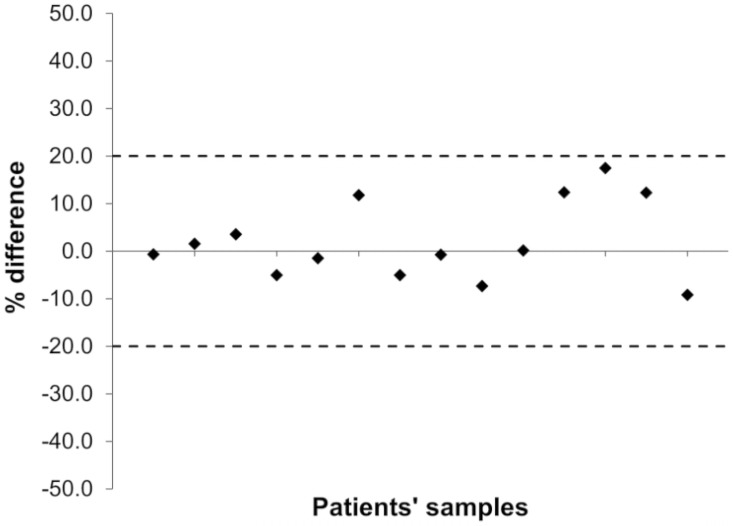
Incurred samples reanalysis: Percentage difference between the first and the second analysis for 14 plasma samples from 6 patients. The dotted lines represent the ±20% deviation limits imposed by EMA and FDA guidelines.

This new quantification method was also reproducible, as demonstrated by the % difference between the two measurements of 14 plasma samples from 6 patients treated with LENVA and analyzed in two different working days. The % difference was between -9.20% and 17.5% (Fig 3), within the ±20% requirements of FDA and EMA guidelines [14, 15].

Even if the ISR is not requested by FDA guideline, we considered it an important test to verify the method reproducibility in “real” samples. Anyway, only the method proposed by Ye et al. [13] performed the ISR on 24 clinical samples with a very good %difference (-4.5% to 3.1%).

#### Application of the method to clinical samples and reproducibility

The presented method was used to successfully quantify 22 plasma samples from 6 patients affected by HCC, treated with LENVA, and recruited in the ongoing above-mentioned analytical study. The patients’ characteristics and the drug dosage are reported in Table 6.

**Table 6:** Principal demographic and clinical patients’ characteristics.

Patients characteristics	N
**Sex**	4males
	2 females
**Mean age (range)**	74 (61–82) years
**Therapy**	6 samples at 4 mg/day
	15 samples at 8 mg/day
	1 sample at 12 mg/day

**Fig 4:**
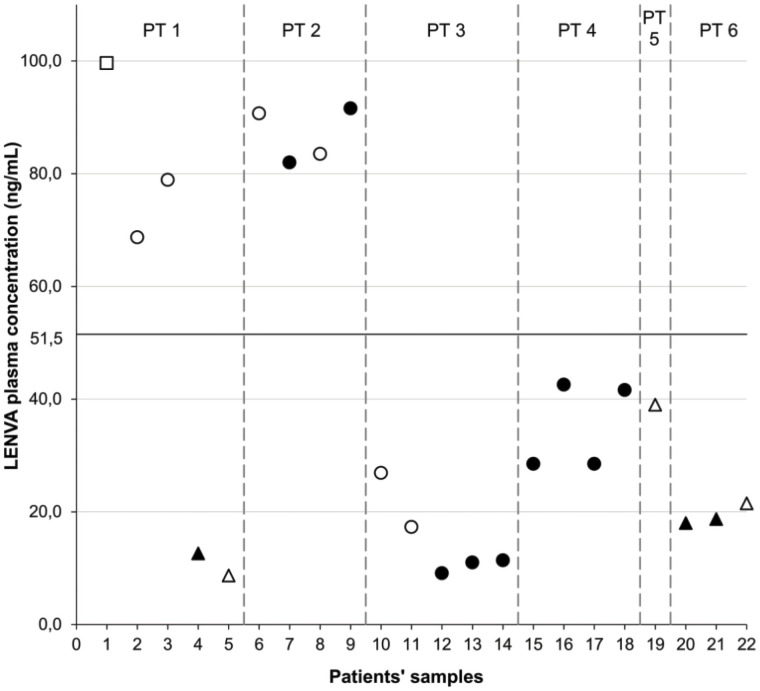
Graphical representation of LENVA concentrations in patients’ plasma samples. (□) corresponds to sample at the dose of 12 mg/day of LENVA collected not at the C_min_; (●) 8 mg/day at the C_min_ and (○) not at the C_min_; (▲) 4 mg/day at the C_min_ and (Δ) not at the C_min_. The line at 51.5 ng/mL corresponds to the mean C_min_ reported in the literature as proposed TDM threshold.

Blood samples were taken between 1.5 and 25.5 h from the last drug assumption. The concentrations found in the samples are reported in Fig 4 and S3 Table. All the samples collected were at the steady-state. The linear range of the calibration curve has demonstrated to be suitable for clinical application since all the quantified samples were within the LLOQ and ULOQ. From the few samples collected no conclusive considerations can be drawn, however, a certain inter-patients variability in drug concentration can be hypothesized by comparing samples of patients treated at the same dosage and collected at comparable times. For instance, considering patients treated with LENVA at 8 mg/day, five samples (7, 9, 12–18), collected around the C_min_ (at 24±1.5 h), displayed a concentration value that spans from 9.1 ng/mL (sample 12) to 91.6 ng/mL (sample 9) and just two of them were above the proposed C_min_ target of 51.5 ng/mL; three samples (2, 10 and 11) collected at about 17(±0.5) h since last drug intake showed a comparable variability (17.3–68.7 ng/mL), while two samples (2 and 7) collected at 4.50 h from last drug administration, exhibited a lower variability (90.7 ng/mL and 78.9 ng/mL). As regards the dosage of 4 mg/day, the concentration of four samples from 2 patients collected approximately at the C_min_ (24±1.5 h) can be described: within patient variability was low, while mean concentration between the two patients is slightly different (10.6 ng/mL vs 18.3 ng/mL).

Generally, a certain intra-patient stability of concentrations was observed when comparing multiple samplings at the same drug dosage collected at comparable times. This observation, even descriptive, corroborates the suitability of LENVA as a candidate for TDM [17, 18].

The wide analytical range of the new proposed method represents an advantage respect the method developed by Janssen et al. [10], that quantified simultaneously other different kinase inhibitors and with a comparable run time (4 min). In fact, 3 out of 22 patients plasma samples that we quantified had a LENVA concentration lower than their LLOQ (10 ng/mL).

## Conclusions

LC-MS/MS analytical methods for TDM application should meet the demands for low sample volumes, simplicity of sample preparation, short run times, reliability, robustness, selectivity, precision, sensitivity, and cost-effectiveness. We developed a new LC-MS/MS method for LENVA quantification in human plasma which required just 100 μL of patient’s plasma. Samples are rapidly processed by PP with the addition of 5 volumes of methanol containing LENVA-D_4_ as IS. Finally, the method has a very short run-time of just 4 min, an easy sample preparation based on PP and ISR proved its robustness and reliability.

The proposed method was characterized by a wide analytical range (0.50–2000 ng/mL) which not only properly covered the therapeutic plasma concentrations of HCC patients, but also makes it applicable for PK investigations in patients affected by other pathologies which require the use of LENVA at higher doses (24 mg/day), in which the mean value of C_max_ at steady state ranged from 430–660 ng/mL [19] or even for hypothetical future applications whether higher doses of LENVA may be investigated. Moreover, the method fully complied with the most recent requirements of both EMA and FDA guidelines for the validation of bioanalytical assays [14, 15]. Even if this fast and simple LC-MS/MS quantification method is suitable for TDM application in clinical practice, a further improvement could be represented by the use of dried blood spot (DBS) as matrix for LENVA quantification. In fact, the use of DBS could, for instance, increase patient’s compliance, reduce analysis costs and facilitate samples storage. Thus, the development of a LC-MS/MS method based on DBS matrix could be of considerable interest in this context. The proposed method can be used as reference assay to clinically validate the DBS-based method [20].

## Supporting information

S1 Table Short and long-term stability with precision (CV%) and accuracy% obtained for LENVA. (DOCX)

S2 Table Precision (CV%) and accuracy % data of LENVA dilution integrity in human plasma. (DOCX)

S3 Table LENVA concentrations found in 22 plasma samples from 6 patients. (DOCX)
