# Supplementary material for: A rapid, simple and sensitive LC-MS/MS method for lenvatinib quantification in human plasma for therapeutic drug monitoring
Source: PLoS One. 2021 Oct 26;16(10):e0259137. doi: 10.1371/journal.pone.0259137 (PMC8547652; doi:10.1371/journal.pone.0259137)
Supplement: S1 Table — (DOCX) [file pone.0259137.s001.docx]

**S1 Table. Short and long-term stability with precision (CV%) and accuracy % obtained for LENVA.**

| **Short term stability of LENVA** | | | | |
| --- | --- | --- | --- | --- |
|  | **Nominal concentration (ng/mL)** | **Mean ± SD (ng/mL)** | **CV%** | **Accuracy %** |
| **4 h at RT** | 1.50 | 1.56 ± 0.17 | 10.9 | 104.0 |
|  | 75.0 | 81.7 ± 2.11 | 2.6 | 108.9 |
|  | 1500 | 1427 ± 30.6 | 2.1 | 95.1 |
| **3’ FTC** | 1.50 | 1.43 ± 0.03 | 1.8 | 95.6 |
|  | 75.0 | 77.4 ± 1.16 | 1.5 | 103.2 |
|  | 1500 | 1397 ± 87.4 | 6.3 | 93.1 |
| **94 h at 4°C** | 1.50 | 1.39 ± 0.06 | 4.4 | 92.7 |
|  | 75.0 | 76.7 ± 3.50 | 4.6 | 102.3 |
|  | 1500 | 1340 ± 45.8 | 3.4 | 89.3 |
| **Long-term stability of LENVA** | | | | |
|  | **Nominal concentration (ng/mL)** | **Mean ± SD (ng/mL)** | **CV%** | **Accuracy %** |
| **315 days at -80°C (plasma)** | 1.50 | 1.54±0.06 | 4.1 | 102.9 |
|  | 75.0 | 75.3±2.07 | 2.7 | 100.4 |
|  | 1500 | 1315.0±7.07 | 0.5 | 87.7 |
| **174 days at -80°C (DMSO)** | 1.50 | 1.63±0.05 | 2.9 | 108.9 |
|  | 75.0 | 82.1±0.64 | 0.8 | 109.4 |
|  | 1500 | 1436.7±80.8 | 5.6 | 95.8 |
| **174 days at**  **-20°C (methanol)** | 1.50 | 1.58±0.05 | 2.9 | 105.3 |
|  | 75.0 | 73.5±3.21 | 4.4 | 98.0 |
|  | 1500 | 1336.7±20.8 | 1.6 | 89.1 |

FTC: freeze/thaw cycles; RT: room temperature.
