# Supplementary material for: A rapid, simple and sensitive LC-MS/MS method for lenvatinib quantification in human plasma for therapeutic drug monitoring
Source: PLoS One. 2021 Oct 26;16(10):e0259137. doi: 10.1371/journal.pone.0259137 (PMC8547652; doi:10.1371/journal.pone.0259137)
Supplement: S2 Table — (DOCX) [file pone.0259137.s002.docx]

S2 Table. Precision (CV%) and accuracy % data of LENVA dilution integrity in human plasma.

| **LENVA (N=5)** | | | |
| --- | --- | --- | --- |
| **Nominal concentration (ng/mL)** | **Mean ± SD (ng/mL)** | **CV%** | **Accuracy %** |
| 30.0 | 30.6 ± 1.2 | 4.0 | 102 |
| 300 | 300 ± 10.6 | 3.5 | 99.9 |
