# Supplementary material for: A rapid, simple and sensitive LC-MS/MS method for lenvatinib quantification in human plasma for therapeutic drug monitoring
Source: PLoS One. 2021 Oct 26;16(10):e0259137. doi: 10.1371/journal.pone.0259137 (PMC8547652; doi:10.1371/journal.pone.0259137)
Supplement: S3 Table — (DOCX) [file pone.0259137.s003.docx]

**S3 Table.** **LENVA concentrations found in 22 plasma samples from 6 patients.**

| **Sample** | **Patient** | **LENVA dose (mg/day)** | **Hours from last intake (hh:mm)** | **LENVA concentration (ng/mL)** |
| --- | --- | --- | --- | --- |
| 1 | 1 | 12 | 04:37 | 99.6 |
| 2 | 1 | 8 | 17:35 | 68.7 |
| 3 | 1 | 8 | 04:25 | 78.9 |
| 4 | 1 | 4 | 24:45 | 12.6 |
| 5 | 1 | 4 | 22:25 | 8.70 |
| 6 | 2 | 8 | 04:30 | 90.7 |
| 7 | 2 | 8 | 23:20 | 82.0 |
| 8 | 2 | 8 | N/A | 83.5 |
| 9 | 2 | 8 | 24:23 | 91.6 |
| 10 | 3 | 8 | 17:32 | 26.9 |
| 11 | 3 | 8 | 16:35 | 17.3 |
| 12 | 3 | 8 | 23:55 | 9.10 |
| 13 | 3 | 8 | 24:45 | 11.0 |
| 14 | 3 | 8 | 25:15 | 11.4 |
| 15 | 4 | 8 | 23:50 | 28.5 |
| 16 | 4 | 8 | 24:15 | 42.6 |
| 17 | 4 | 8 | 23:15 | 28.5 |
| 18 | 4 | 8 | 24:03 | 41.6 |
| 19 | 5 | 4 | 12:45 | 39.0 |
| 20 | 6 | 4 | 23:20 | 18.0 |
| 21 | 6 | 4 | 24:45 | 18.7 |
| 22 | 6 | 4 | 01:25 | 21.5 |

N/A: not applicable
